# Supplementary material for: Transmembrane Protein LMxysn_1693 of Serovar 4h Listeria monocytogenes Is Associated with Bile Salt Resistance and Intestinal Colonization
Source: Microorganisms. 2022 Jun 21;10(7):1263. doi: 10.3390/microorganisms10071263 (PMC9320622; doi:10.3390/microorganisms10071263)
Supplement: Supplementary file 1 [file microorganisms-10-01263-s001.zip › microorganisms-1756861-supplementary.pdf]

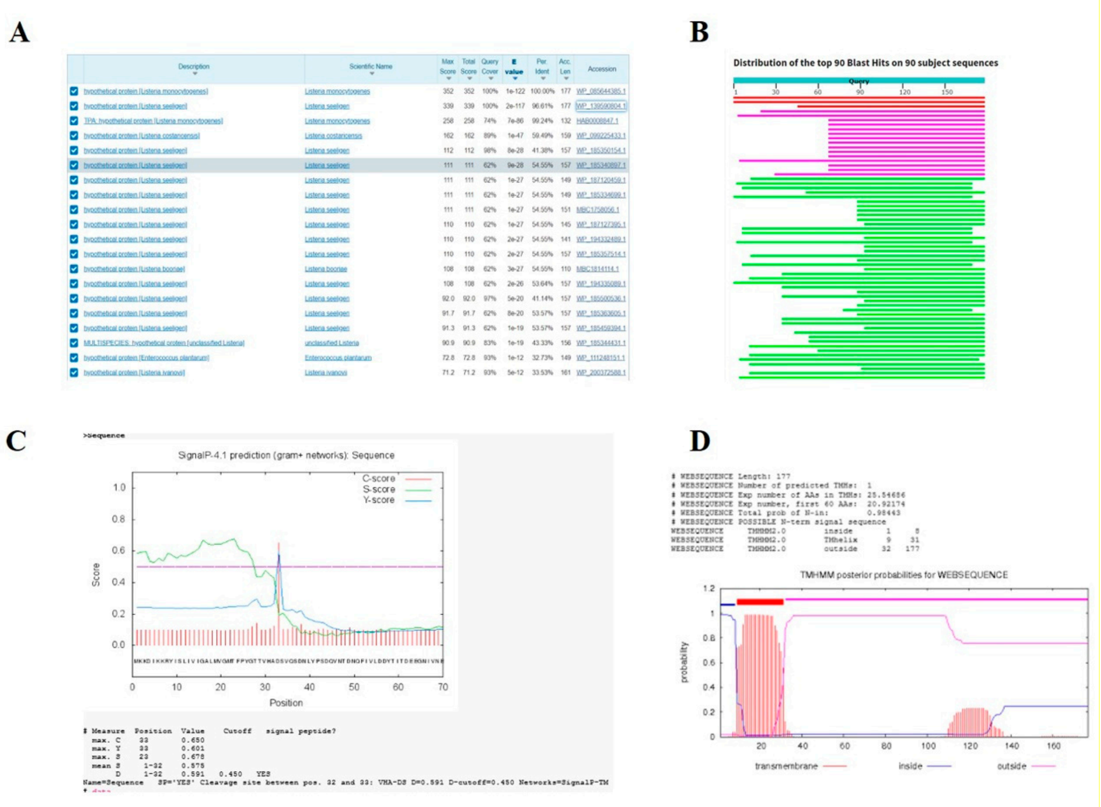

Supplementary Figure S1. Bioinformatics prediction of LMxysn_*1693* deletion and reversion. Homology analysis of the protein encoded by the LMxysn_*1693* using the Blast website (A and B). The signal peptide and membrane expansion structure of the protein encoded by the LMxysn_*1693* gene were analyzed using the SingnalP-6.0 website (C and D). The protein encoded by the LMxysn_*1693* gene is predicted as a hypothetical protein with a signal peptide at the N-terminus and transmembrane once.

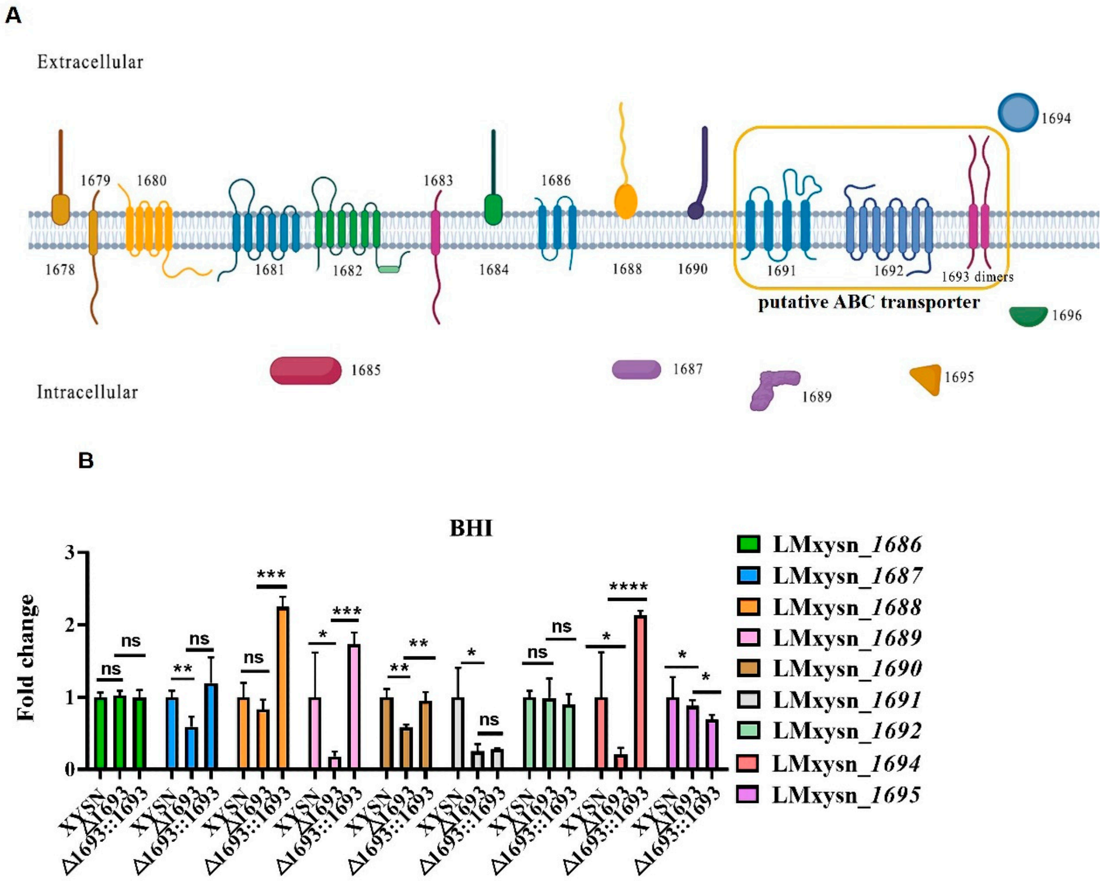

Supplementary Figure S2. Bioinformatics prediction and detecting expression levels of GI-7. The cellular localization and functional predication of the proteins encoded by 20 genes distribution in the GI-7 (A). The expression levels of genes in the GI-7 were detected with LMxysn_*1693* deletion strain by qRT-PCR (B) Error bars represent SD, n = 3 independent experiments. Statistical analysis was performed using Student's t-test: ns, none significance, $^{*}p < 0.05$, $^{**}p < 0.01$, $^{***}p < 0.001$, $^{****}p < 0.0001$.

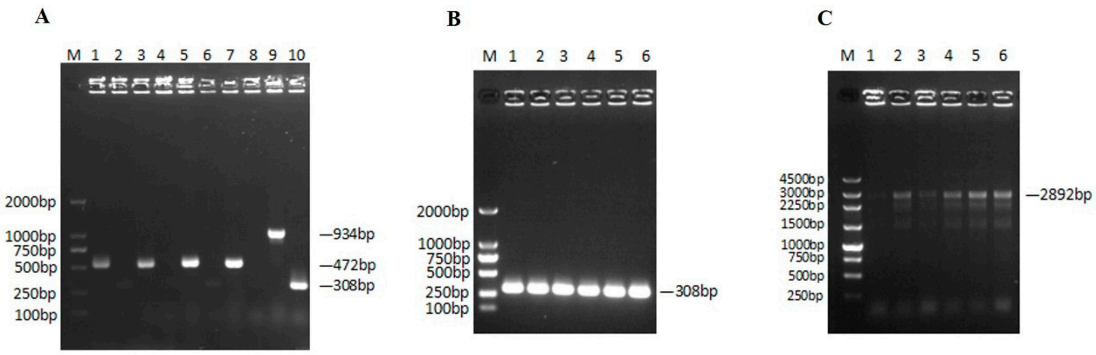

Supplementary Figure S3. The construction of LMxysn_*1693* deletion and recovery strains. PCR identification of XYSNΔ*1693* using primers outside the homology arms of the LMxysn_*1693* gene and inside primers inside the gene (A). Lane M is Marker DL2000, lanes 1, 3, 5, and 7 are PCR amplification results of outer primers, and the band size is about 472 bp; lanes 2, 4, 6, and 8 are PCR amplification results of inner primers, and no band is amplified Obtained, lane 9 is the identification result of XYSN using the outer primer, and lane 10 is the identification result of XYSN using the inner primer. PCR identification of XYSNXYSNΔ*1693*::*1693* using primers outside the homology arms of the LMxysn_*1693* gene and inside primers inside the gene (B and C). Lane M is Marker DL2000 and Marker DL5000 respectively, the size of the amplified band of inner primers is 308bp (B), and the size of the amplified band of outer primers is 2892bp (C).

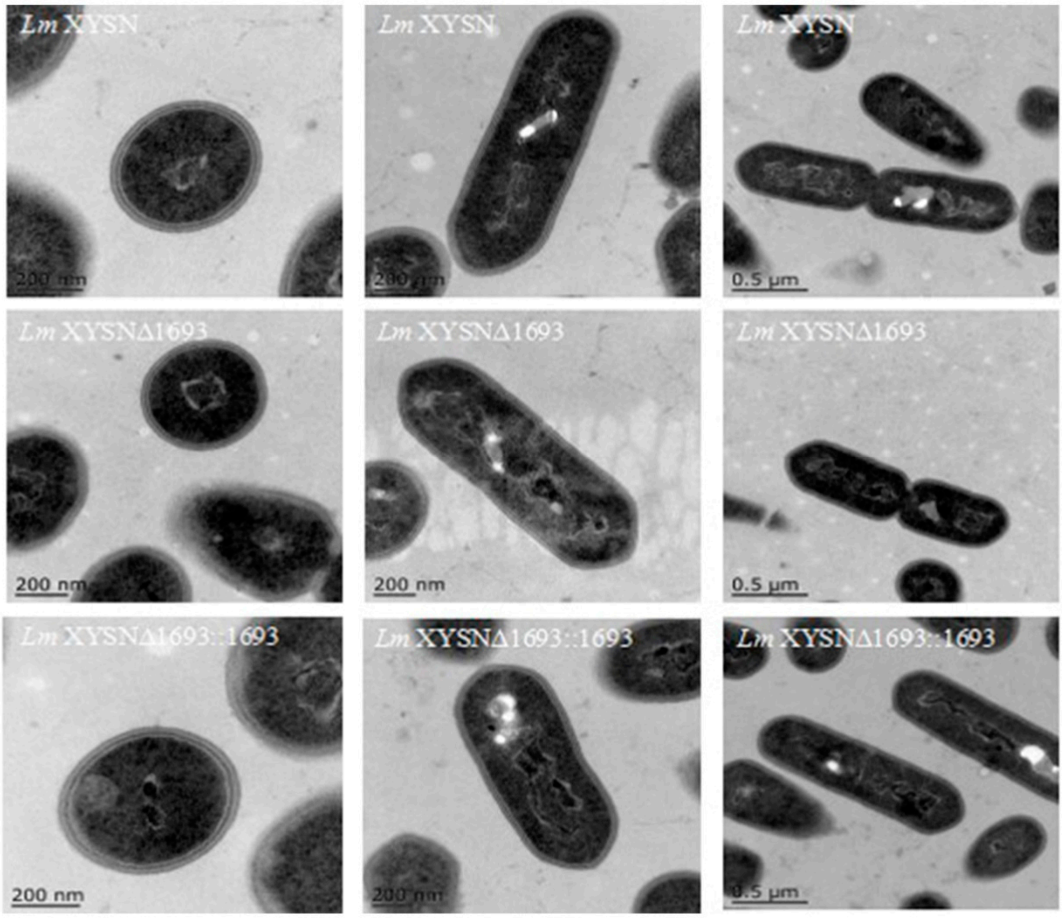

Supplementary Figure S4. Transmission electron microscopic images depicting intact cell wall structures of XYSN, XYSNΔ*1693* and XYSNΔ1095::1095.

Supplementary Table S1. Primers for construction of mutant and complement strains used in this study

Primers for construction of mutant and complement strains

Primer Code	Primers Sequences (5'-3')
**Primer Code**	**Primers Sequences (5′-3′)**
LMxysn_1693 P1	TTGTAAAACGACGGCCAGTGAATTCATCACTTTGGATTCTGCCTTT
LMxysn_1693 P2	AAGAGGGAGACAACGATGAAGAAAGAGTATGTTGGAACCTACACCTAT
LMxysn_1693 P3	ATAGGTGTAGGTTCCAACATACTCTTTCTTCATCGTTGTCTCCCTCTT
LMxysn_1693 P4	TGCATGCCTGCAGGTCGACTCTAGATTATGCTTCTAAACCGTCTGC
LMxysn_1693 N1	CACCTTTTACGATTACTTTCACAC
LMxysn_1693 N2	GGTGGGTATGACCTTTCCTTATGG
LMxysn_1693 W1	TTACGGGATTTGTTGTATATCGAAG
LMxysn_1693 W2	ATAATAACTAACCCACTAACCACCT
LMxysn_1693H P1	TGTAAAACGACGGCCAGTGAATTCTGTGTTTTTCGTTATCAATGTGG
LMxysn_1693H P2	ATGTAGATAGAATTCCAGTATAACAATATCAATTCAAGTGCGTTT
LMxysn_1693H P3	AAACGCACTTGAATTGATATTGTTATACTGGAATTCTATCTACAT
LMxysn_1693H P4	GCATGCCTGCAGGTCGACTCTAGATTGGTCAGATAAAGACGTAGTAGG
LMxysn_1693H N1	CACCTTTTACGATTACTTTCACAC
LMxysn_1693H N2	GGTGGGTATGACCTTTCCTTATGG
LMxysn_1693H W1	TTCTCTACCGCACTTTTCTTCCCC

Supplementary Table S2. Primers for quantitative real-time PCR detection used in this study

Primers for quantitative real-time PCR detection

Primer Code	Primers Sequences (5'-3')	Primer Code	Primers Sequences (5'-3')
gyrb P1	AGACGCTATTGATGCCGATGA	gyrb P2	GTATTGCGCGTTGTCTTCGA
bsh P1	TAATGGAGACAGCGGTAAAT	bsh P2	TTATTCCGTGGATTCTTGGT
sigB P1	TCGGATGGAAGTACGATTAC	sigB P2	TCTTCTGTTCTCGCTCATCT
brtA P1	TTATTACGGCGTTCACTTAT	brtA P1	TTATTACGGCGTTCACTTAT
prfA P1	AGCCAACCGATGTTTCTGT	prfA P2	TATTAGCGAGAACGGGACC
ami P1	TGGCACCTCCGCTACTATGA	ami P2	CCGTTGCAGTCCCATCTC
actA P1	TATGCGTGCGATGATGGT	actA P2	ACCTCGCTTGACTGCTCT
hly P1	AAATGCCACTAAATCAAACG	hly P2	CACTGTAAGCCATTTCGTCA
LMxysn_1686 P1	CAGTGGCACATCAATACCCT	LMxysn_1686 P2	TTGGGTTTGTATTAAGTGGT
LMxysn_1687 P1	GTTCGGTTGCTTCATAGGTT	LMxysn_1687 P2	ACCAAACGAAGATTTCACCT
LMxysn_1688 P1	TGGCGATGTTTCCTGTATTT	LMxysn_1688 P2	CCAAAAGAAGGAATCGAAAT
LMxysn_1689 P1	CCTTGTCCTTAGAATGTTCC	LMxysn_1689 P2	TCACGAACGAAGAACAAAAC
LMxysn_1690 P1	CATCTGGCAAAGCATCTGTA	LMxysn_1690 P2	CATTCCTACGAGCGTATTGG
LMxysn_1691 P1	TGTGATTTTACTCCCGATGT	LMxysn_1691 P2	CATTATTTGGTTTTGGAGGA
LMxysn_1692 P1	TCCAAATTGTTATCAGACGC	LMxysn_1692 P2	AGGTAATTTTCTCCCAACAG
LMxysn_1694 P1	AAAGTCCTTCAAATAAACCAT	LMxysn_1694 P2	TCTGAAAGTGTTGAAGGAGGA
LMxysn_1695 P1	ACTTTGTATTGATAAGCATCTCCA	LMxysn_1695 P2	CGTAGTAGGAACGGTAGGTAAAGG

Supplementary Table S3. Determination the minimum inhibitory concentration of Lm

Categories	XYSN (μg/mL)	XYSNΔ*1693* (μg/mL)	XYSNΔ*1693::1693* (μg/mL)	ATCC25923 (μg/mL)
**PG**	64	64	64	64
**CL**	32	32	32	32
**CHL**	1	2	1	1
**NVA**	1	1	1	2
**AMP**	1	1	1	1

The effect of LMxysn_*1693* gene deletion on bacterial resistance was detected by the minimum inhibitory concentration experiment using different antibiotics. The antibiotics used in this experiment were penicillin G, vancomycin and ampicillin (acting on the cell wall), polymyxin B (acting on the cell membrane), and

chloramphenicol (acting on the ribosomal 50S subunit).

Supplementary Table S4. Physiological and biochemical characteristics of Lm

	AMY	PIPLC	dXYL	ADH1	BGAL	AGLU	APPA	CDEX	AspA	dRAF	O129R
XYSN	+	+	−	−	−	+	−	+	−	−	+
XYSNΔ*1693*	+	+	−	−	−	+	−	+	−	−	+
XYSNΔ*1693::1693*	+	+	−	−	−	+	−	+	−	−	+
	BGAR	AMAN	PHOS	LeuA	ProA	BGURr	AGAL	PyrA	BGUR	SAL	SAC
XYSN	−	+	−	−	−	−	−	−	−	+	−
XYSNΔ*1693*	−	+	−	−	−	−	−	−	−	+	−
XYSNΔ*1693::1693*	−	+	−	−	−	−	−	−	−	+	−
	AlaA	TyrA	dSOR	URE	POLYB	dGAL	dRIB	ILATk	LAC	dTRE	ADH2s
XYSN	−	+	−	−	+	+	+	−	−	+	−
XYSNΔ*1693*	−	+	−	−	+	+	+	−	−	+	−
XYSNΔ*1693::1693*	−	+	−	−	+	+	+	−	−	+	−
	NAG	dMAL	BACI	NOVO	NC6.5	dMAN	dMNE	MBdG	PUL	OPTO	
XYSN	+	+	+	+	+	−	+	+	−	+	
XYSNΔ*1693*	+	+	+	+	+	−	+	+	−	+	
XYSNΔ*1693::1693*	+	+	+	+	+	−	+	+	−	+	

Biochemical determination was conducted with wild strains, deletion strains and restored strains, - means negative result, + means positive result, which is consistent with the description in the instructions.
